# Spinopelvic Dissociation: Comparison of Outcomes of Percutaneous versus Open Fixation Strategies

**DOI:** 10.1155/2018/5023908

**Published:** 2018-04-10

**Authors:** Jeffrey M. Pearson, Thomas E. Niemeier, Gerald McGwin, Sakthivel Rajaram Manoharan

**Affiliations:** ^1^Department of Orthopedic Surgery, University of Alabama at Birmingham, Birmingham, AL, USA; ^2^Department of Epidemiology, University of Alabama at Birmingham, Birmingham, AL, USA

## Abstract

**Introduction:**

Spinopelvic dissociation injuries are historically treated with open reduction with or without decompressive laminectomy. Recent technological advances have allowed for percutaneous fixation with indirect reduction. Herein, we evaluate outcomes and complications between patients treated with open reduction versus percutaneous spinopelvic fixation.

**Methods:**

Retrospective review of patients undergoing spinopelvic fixation from a single, level one trauma center from 2012 to 2017. Patient information regarding demographics, associated injuries, and treatment outcome measures was recorded and analyzed. All fractures were classified via the AO Spine classification system.

**Results:**

Thirty-one spinopelvic dissociations were identified: 15 treated with open and 16 with percutaneous techniques. The two treatment groups had similar preoperative characteristics including spinopelvic parameters (pelvic incidence and lumbar lordosis). Compared to open reduction internal fixation, percutaneous fixation of spinopelvic dissociation resulted in statistically significantly lower blood loss (171 cc versus 538 cc; *p* = 0.0013). There were no significant differences in surgical site infections (*p* = 0.48) or operating room time (*p* = 0.66).

**Conclusion:**

Percutaneous fixation of spinopelvic dissociation is associated with significantly less blood loss. Treatment outcomes in terms of infection, length of stay, operative cost, and final alignment between the open and percutaneous group were similar.

## 1. Introduction

**Figure 1:**
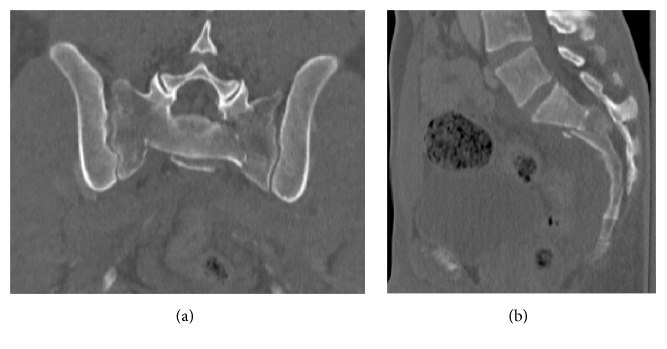
Coronal (a) and midsagittal (b) preoperative CT scan. Note the bilateral sacral fractures with horizontal S2 fracture with a 37-degree kyphotic deformity.

**Figure 2:**
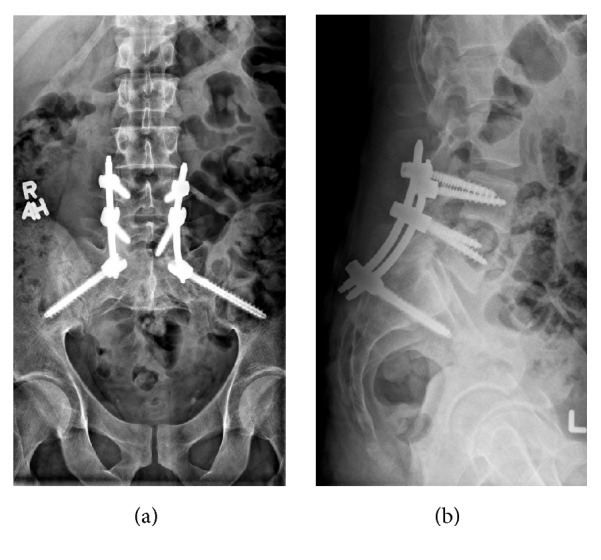
AP (a) and lateral (b) radiographs of patient seen in Figure 1 at 16 months postoperatively treated with percutaneous fixation. Note the resolution of kyphotic deformity with consolidation of fracture lines.

Spinopelvic dissociation or U type sacrum fracture is a rare injury that involves a transverse sacral fracture pattern and can be associated with a high rate of neurologic injury, up to 57% (Figure 1) [1–4]. In large case series, spinopelvic dissociative injuries account for only 2.9% of all pelvic ring traumas [5, 6]. Traditional treatment methods involving open reduction with internal fixation have been observed to have high rates of postoperative infections up to 14–16% [7–10]. In the last decade, percutaneous fixation (Figure 2) of these injuries has gained popularity with reported improved clinical outcomes [11, 12].

Herein, we critically evaluate results of spinopelvic dissociations treated with either percutaneous or open reduction. To our knowledge, this is the largest series of patients with spinopelvic dissociation treated surgically. We hypothesized that percutaneous fixation of spinopelvic dissociation using minimally invasive fusion from lumbar four or five to pelvis would result in shorter operative time, fewer postoperative transfusions, and decreased estimated blood loss while adequately reestablishing spinopelvic parameters.

## 2. Methods

A retrospective review was conducted on all operatively managed spinopelvic dissociations treated between January 2012 and March 2017 at a single, level one trauma center. Institutional review board approval was obtained. Inclusion criteria were patients over 18 years of age with diagnosed spinopelvic dissociation based on pelvic X-rays and CT scans. Patients with preoperative lower lumbar, sacral, or pelvic hardware were excluded. Patient characteristics and demographics were collected for all patients including age, gender, mechanism of injury, associated injuries, neurological injury, and tobacco use. The injuries were classified according to the Arbeitsgemeinschaft für Osteosynthesefragen (AO) sacrum classification. Pelvic X-rays were also subclassified based on modifiers M3 being anterior pelvic ring injury and M4 sacroiliac joint injury [13].

All surgeries were performed by fellowship-trained spine, orthopedic surgeons during this initial inpatient stay. The decision of open versus percutaneous fixation was nonrandomized and at the judgment of the attending surgeon. This was usually based on associated injuries such as soft tissue near a possible incision site, body habitus, and surgeon training. Younger trained surgeons tended to perform percutaneous procedures while older ones tended to perform traditional open reduction techniques. All percutaneous surgeries used indirect reduction and four or five percutaneous lumbar pedicle screws with iliac fixation as described by Wang et al. [11]. Percutaneous transsacral fixation was not used in any of the patients. Neurological dysfunction (cauda equina or sacral nerve root disruption) was treated with either laminectomies or fracture reduction with indirect decompression.

The surgical procedure began with standard spinal monitoring and general anesthesia. The patients were positioned prone on an open top table with large bumps under the thighs to accentuate an extension force on the legs. This high extension was our main reduction maneuver in the surgical procedure. Bilateral L5 pedicle screws were placed under fluoroscopic guidance with Jamshidi needles, and these were also used for the iliac fixation. Initial placement of the needle for iliac fixation was just ventromedial to the PSIS. From this point using a teardrop view, the Jamshidi needle was guided down between the inner and the outer tables of the pelvis. Following placement of the needle, inlet, outlet, and lateral views were obtained to ensure no violation of the sciatic notch.

In-hospital clinical outcomes were measured and included surgical site infection, transfusions within 72 hours postoperatively, operative time, and estimated blood loss. After discharge, patients were followed up at routine intervals as outpatients. Postoperative complications including hardware irritation and spinopelvic parameters (pelvic incidence and lumbar lordosis) were recorded for all patients. All patients were made weight bearing as tolerated unless other lower extremity fractures prohibited this.

Demographic, clinical, and outcome measures were compared between the fixation groups using Fisher's exact test and Student's *t*-test for categorical and continuous variables, respectively. *p* values ≤ 0.05 (two-sided) were considered statistically significant.

## 3. Results

Thirty-one patients with spinopelvic dissociation were identified at our institution from 2012 to 2017. The cohort consisted of 15 patients treated with open reduction and lumbopelvic fixation and 16 with indirect reduction and percutaneous lumbopelvic fixation. Age, sex, and tobacco use were similar between the two groups (*p* > 0.05) (Table 1). Outpatient follow-up was on average 7.8 months for the open group and 9.9 months for the closed group (*p* = 0.50). Both groups included three patients with preoperative symptoms of cauda equina.

Injury patterns were classified based on the AO sacrum classification system [13]. Our results can be found in Table 2. Fractures were classified as nondisplaced sacral U type variant (C0), sacral U type variant without posterior instability (C1), bilateral complete type B fractures without transverse fracture (C2), and displaced U type sacrum fracture (C3). In addition to these categories, fractures were also classified based on modifiers of anterior pelvic ring injury (M3) and sacroiliac joint injury (M4). Of the 15 open procedures, 5 were C0, 1 was C2, and 9 were C3. Of these 15, a total of 6 were M3 modifiers signifying anterior pelvic ring injury. Of the 16 percutaneous procedures, 8 were classified as C0 and 8 were C3. Five from the C0 percutaneous subgroup had M3 modifiers. Of the C3 group, 2 had an anterior ring injury (M3), 1 had a posterior sacroiliac joint disruption (M4), and a total of 2 had both modifiers (M3, M4). This information can be found in Table 2.

Associated injuries are shown in Table 3. The cohort of 31 patients included 18 (58%) with long bone fractures (8 in the open and 10 in the closed group) that prompted surgical treatment. Other associated injuries included closed head injuries (42%), acetabular fractures (39%), and other spine fractures (48%).

Postoperative outcomes are shown in Table 4. Mean intraoperative blood loss was significantly higher (*p* = 0.0013) in the open reduction (538 cc) group in comparison to the percutaneous group (171 cc). Transfusions within 72 hours postoperatively were seen in seven patients (43%) treated with percutaneous fixation and in three patients (20%) treated with open reduction (*p* = 0.25). Average length of stay for the open group was 14.9 days and 17.5 days in the percutaneous group (*p* = 0.57). OR cost for the percutaneous procedure averaged at $83,705.80 per case and $63,963.13 for the open procedure (*p* = 0.29).

**Figure 3:**
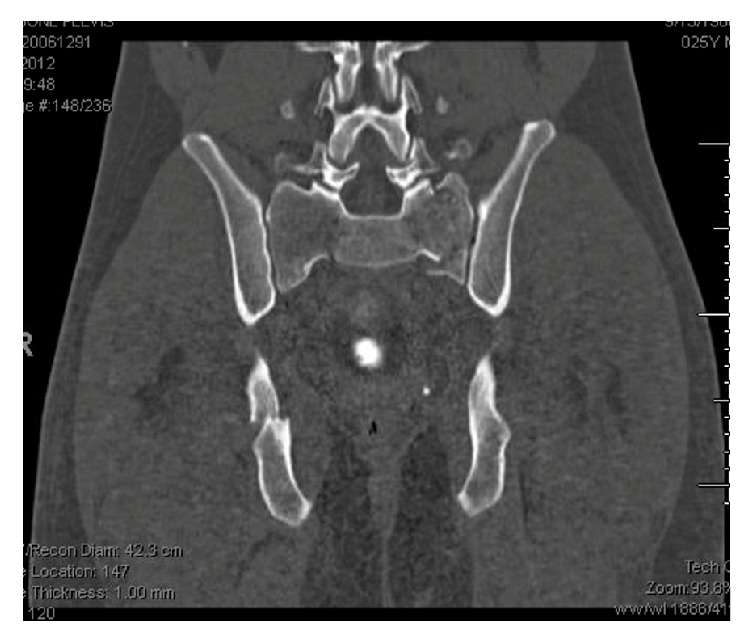
Preoperative coronal CT scan of a patient with spinopelvic dissociation.

**Figure 4:**
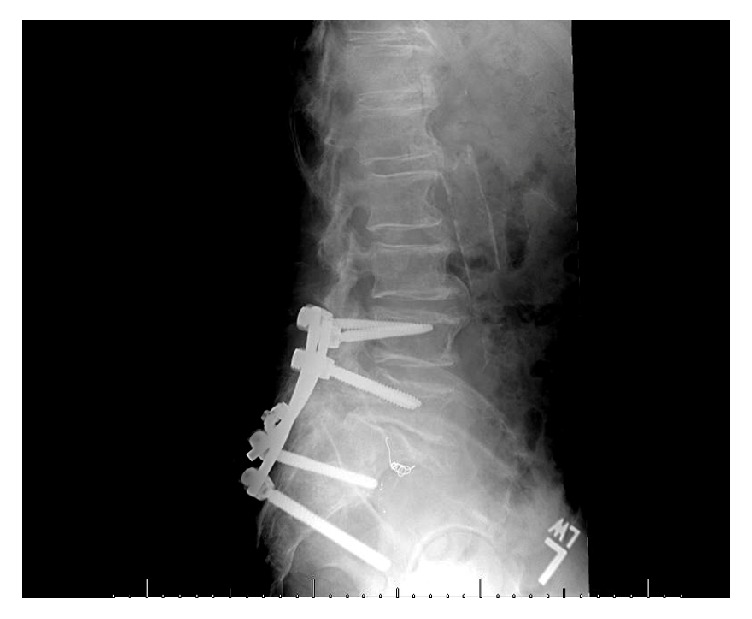
Postoperative films of patient treated with open reduction internal fixation of spinopelvic dissociation.

Postoperatively, spinopelvic radiographic parameters (lumbar lordosis and pelvic incidence) were similar between the two groups (Table 3). Average lumbar lordosis was 54.1 degrees in the open group and 53.8 degrees in the percutaneous group (*p* = 0.96). Pelvic incidence was 65.8 degrees in the open group and 64.7 degrees in the closed group (*p* = 0.84). No surgical site infections were seen in the percutaneous treatment cohort, whereas one infection was observed with the open reduction cohort (*p* = 0.48). A comparable amount in each group underwent hardware removal at a later date; 40% (6/15) in the open group and 31% (5/16) in the closed group underwent hardware removal (*p* = 0.72). Figures 3 and 4 show another example of patients in our cohort. This shows pre- and postoperative images for fixation with open reduction internal fixation.

## 4. Discussion

Modern technologic advances in orthopedic instrumentation have allowed for the adoption of percutaneous techniques to treat spinopelvic dissociation. Historic treatment with open reduction with lumbopelvic fixation of spinopelvic dissociation has been associated with a high infection rate [9, 10]. Schildhauer et al. observed an infection rate of 16% with open reduction and internal fixation of spinopelvic dissociations [8]. With current minimally invasive strategies, percutaneous instrumentation has been shown to have low rates of wound complication and decreased blood loss [12].

In the current study, we compared surgical as well as postoperative outcomes of open versus percutaneous fixation in a cohort of 31 patients. To our knowledge, this is the largest study to be reported on patients with spinopelvic dissociation treated surgically [9, 12, 14]. Through utilizing indirect reduction techniques as described by Nork et al., we were able to show satisfactory fracture reduction with equivalent (*p* = 0.96, 0.84) postoperative lumbar lordosis (LL) and pelvic incidence (PI) in both treatment groups [5, 15]. The limitation of our study is that spinopelvic measures were primarily evaluated compared to the actual reduction of the fracture site. Though there is individual variability in the spinopelvic parameters, studies have shown that it can be used as a surrogate of fracture reduction in the sagittal plane and the primary driver of clinical outcome [16].

In the current study, percutaneous fixation of spinopelvic dissociation showed a statistically lower intraoperative blood loss compared to open reduction internal fixation (171 cc versus 528 cc, *p* = 0.0013) and a lower rate of postoperative infections (1 versus 0, *p* = 0.483). Blood loss is difficult to analyze as a sole outcome when associated injuries are not taken into account. Aside from traumatic brain injury and closed head injury, the closed cohort had more associated injuries than the open group (Table 4). This discrepancy in preoperative patient characteristics likely explains the increased length of stay in our percutaneous group compared to the cohort. Despite the procedure being one that results in less blood loss and shorter operative time, the percutaneous patient population had more injuries on average than the open group, likely resulting in a longer hospital stay. Our results correlate with the literature seen with minimally invasive spine (MIS) that emphasized improved short-term clinical outcomes with percutaneous technology [17–20]. Meta-analysis by Phan and colleagues confirmed no excessive screw malposition, decreased infection, and decreased hospital stay with percutaneous spinal techniques [20]. Furthermore, given the severe nature of the trauma sustained by patients with spinopelvic dissociation, these patients are undergoing many procedures for various injuries such as long bone fractures. In settings such as this, the benefits of minimally invasive procedures cannot be overstated.

One surgical site infection was observed in the cohort. The patient was involved in an ATV accident and sustained a type C3, M3 (AO Spine classification) spinopelvic dissociation in addition to anterior pelvic ring disruption with 5 cm of pubic symphysis diastasis. Ten days after a combined anterior pelvic ring and open posterior spinopelvic fixation, the patient underwent debridement with retention of hardware. With prolonged IV antibiotics, the patient progressed to bony union without any further intervention.

Operative time was twenty-nine minutes shorter in the percutaneous group in comparison to the open group. While this relationship does not meet statistical significance, it is of clinical and financial importance. Work by Macario estimates that operating room time per minute in US hospitals costs an average of $62 [21]. In review of our hospitals' cost data, we found that the total cost for the percutaneous group was greater (*p* = 0.29) than of the open treatment group. This increased cost is likely due to many factors such as using new percutaneous implants and intraoperative navigation; however, as itemized cost data were not available, we are unable to pinpoint an exact reason for the difference in cost. Further, we noted that there were decreased operative times in the percutaneous group despite the rising learning curve of the surgeon and the fluoroscopy staff.

Sacral laminectomy and decompression were performed only in patients who had focal compression of sacral nerve roots. In our study, 6 of the 31 displaying bowel and bladder dysfunction did not have decompression. Unlike cauda equina of the lumbar spine, neurological injuries secondary to sacral fractures are not considered neurologic emergencies requiring decompression within 48 hours. Schildhauer and colleagues showed that surgical timing did not correlate with clinical outcomes in patients with cauda equina secondary to spinopelvic dissociation [8]. This was confirmed by Lindahl et al. who additionally showed that laminectomy did not improve bladder or bowel function in patients who underwent decompression [22]. The role of open sacral decompression on ultimate outcome remains unclear and is beyond the scope of the current study. As shown earlier, the reduction of the fracture via percutaneous methods is comparable to that obtained with open reduction internal fixation.  Figure 3 shows the pre- and postoperative imaging for a patient who underwent percutaneous fixation whereas Figure 4 shows the same imaging with an open reduction. As evidenced here, similar reduction can be obtained without the need for an invasive approach.

There are several limitations to this study including those inherent to a retrospective review. First and foremost, selection bias plays a role in all retrospective reviews. The decision to treat injuries, open versus closed, was based on the surgeon's preference and experience. Factors going into choosing which intervention a patient underwent are likely related to physician preference as well as body habitus, overall physiologic health, and underlying comorbidities. The majority of our patient selection was based on the age and training of the surgeon. The two younger physicians with more recent training tended to perform percutaneous techniques whereas the older physicians performed open reduction. The type of surgical skill necessary for both open and closed procedures varies as well. In the percutaneous procedure, one must master positioning of the Jamshidi needle in space while correlating this to the radiographic images. This type of procedure does not involve sacral laminectomy and the fine dexterity required in this aspect of an open approach. The patients reviewed were subject to care under several different surgeons and were nonrandomized to treatment groups. Unfortunately, the retrospective chart review did not include information on the soft tissue condition of the patients. This would have certainly affected the decision-making of the treating team had this been documented in the chart. Given the severe nature of the trauma sustained by these patients, it is inherently difficult to independently evaluate outcomes of a single procedure given their multisystem traumatic injuries. As Table 3 shows, the shear nature of the diversity of these patients and their extensive injuries makes a thorough comparison of these two patient groups difficult. Though it is a large series on an infrequently encountered injury, larger and better powered studies are required to further validate our results. We recognize the relatively short follow-up for this cohort and the need for longer studies to fully evaluate the effects of open versus closed surgical treatment in these patients.

In conclusion, percutaneous fixation of spinopelvic dissociation showed superior outcomes compared to open reduction internal fixation. Percutaneous fixation allowed for a statistically significant decrease in operative blood loss, while also showing a trend of decreasing the operating room time and surgical site infection. Though biomechanically triangular osteosynthesis is superior to stand-alone lumbopelvic fixation, clinically stand-alone fixation without the addition of iliosacral screws results in good outcomes in our series. Percutaneous fixation of spinopelvic dissociation is a less morbid and more expeditious method of pelvic stabilization compared to open reduction internal fixation.

## Figures and Tables

**Table 1:** Preoperative patient characteristics.

	Open (*n* = 15)	Closed (*n* = 16)	*p* value
Age	44.86	37.87	0.3046
Gender	Male: 9 Female: 6	Male: 11 Female: 5	0.7160
Tobacco use	8 (53%)	8 (50%)	0.4589
Cauda equina	3 (20%)	3 (18%)	1.00

**Table 2:** Fracture pattern classification. All fracture patterns were AO sacrum classification type C.

AO Spine	C0 (nondisplaced U type sacrum fracture)				C1 (alternative sacrum U type fracture without posterior instability)				C2 (bilateral complete type B injuries without transverse fracture)				C3 (displaced U type sacrum fracture)			
Modifiers	–	M3	M4	M3, 4	–	M3	M4	M3, 4	–	M3	M4	M3, 4	–	M3	M4	M3, 4
*Open*	5	2			0				1	1			9	3		
*Closed*	8	5			0				0				8	2	1	2

**Table 3:** Associated traumatic injuries.

	Open (*n* = 15)	Closed (*n* = 16)	Total (*n* = 31)
Traumatic brain injury	2 (13%)	1 (6%)	3 (10%)
Closed head injury	9 (60%)	4 (25%)	13 (42%)
Extremity fracture	8 (53%)	10 (63%)	18 (58%)
Anterior pelvic ring fracture	6 (40%)	11 (69%)	17 (55%)
Acetabulum fracture	4 (27%)	5 (31%)	9 (29%)
Thoracic injury	7 (47%)	10 (63%)	17 (55%)
Blunt abdominal injury	4 (27%)	6 (38%)	10 (32%)
Associated spine injury	6 (40%)	9 (56%)	15 (48%)

**Table 4:** Postoperative outcomes on operatively treated patients. Note that blood loss was statistically significantly less in the percutaneous cohort.

	Open (*n* = 15)	Closed (*n* = 16)	*p* value
Pelvic incidence	65.8°	64.68°	0.8413
Lumbar lordosis	54.14°	53.81°	0.9568
Surgical site infection	1 (6.67%)	0 (0%)	0.4839
Transfusion post-op	3 (20%)	7 (43%)	0.2524
Operative time (minutes)	311	282	0.6665
Estimated blood loss (cc)	538	17	0.0013
Hardware removal	6 (40%)	5 (31%)	0.7160
Length of hospital stay (days)	14.91	17.45	0.5696
Length of follow-up (months)	7.75	9.93	0.5042
OR charges	$249,387.95	$347,205.22	0.1837
OR cost	63,963.13	$83,705.80	0.2922
